# A New Mobile Phone-Based Tool for Assessing Energy and Certain Food Intakes in Young Children: A Validation Study

**DOI:** 10.2196/mhealth.3670

**Published:** 2015-04-24

**Authors:** Hanna Henriksson, Stephanie E Bonn, Anna Bergström, Katarina Bälter, Olle Bälter, Christine Delisle, Elisabet Forsum, Marie Löf

**Affiliations:** ^1^Linköping UniversityDepartment of Clinical and Experimental MedicineLinköpingSweden; ^2^Karolinska InstitutetDepartment of Biosciences and NutritionHuddingeSweden; ^3^Karolinska InstitutetDepartment of Medical Epidemiology and BiostatisticsStockholmSweden; ^4^Karolinska InstitutetInstitute of Enviromental MedicineStockholmSweden; ^5^Royal Institute of TechnologySchool of Computer Science and CommunicationStockholmSweden

**Keywords:** cell phone, digital camera, food intake, energy intake, child, DLW, FFQ

## Abstract

**Background:**

Childhood obesity is an increasing health problem globally. Obesity may be established already at pre-school age. Further research in this area requires accurate and easy-to-use methods for assessing the intake of energy and foods. Traditional methods have limited accuracy, and place large demands on the study participants and researchers. Mobile phones offer possibilities for methodological advancements in this area since they are readily available, enable instant digitalization of collected data, and also contain a camera to photograph pre- and post-meal food items. We have recently developed a new tool for assessing energy and food intake in children using mobile phones called the Tool for Energy Balance in Children (TECH).

**Objective:**

The main aims of our study are to (1) compare energy intake by means of TECH with total energy expenditure (TEE) measured using a criterion method, the doubly labeled water (DLW) method, and (2) to compare intakes of fruits and berries, vegetables, juice, and sweetened beverages assessed by means of TECH with intakes obtained using a Web-based food frequency questionnaire (KidMeal-Q) in 3 year olds.

**Methods:**

In this study, 30 Swedish 3 year olds were included. Energy intake using TECH was compared to TEE measured using the DLW method. Intakes of vegetables, fruits and berries, juice, as well as sweetened beverages were assessed using TECH and compared to the corresponding intakes assessed using KidMeal-Q. Wilcoxon matched pairs test, Spearman rank order correlations, and the Bland-Altman procedure were applied.

**Results:**

The mean energy intake, assessed by TECH, was 5400 kJ/24h (SD 1500). This value was not significantly different (*P*=.23) from TEE (5070 kJ/24h, SD 600). However, the limits of agreement (2 standard deviations) in the Bland-Altman plot for energy intake estimated using TECH compared to TEE were wide (2990 kJ/24h), and TECH overestimated high and underestimated low energy intakes. The Bland-Altman plots for foods showed similar patterns. The mean intakes of vegetables, fruits and berries, juice, and sweetened beverages estimated using TECH were not significantly different from the corresponding intakes estimated using KidMeal-Q. Moderate but statistically significant correlations (*ρ*=.42-.46, *P=*.01-.02) between TECH and KidMeal-Q were observed for intakes of vegetables, fruits and berries, and juice, but not for sweetened beverages.

**Conclusion:**

We found that one day of recordings using TECH was not able to accurately estimate intakes of energy or certain foods in 3 year old children.

## Introduction

According to the World Health Organization (WHO), childhood obesity is one of the most serious public health challenges of the 21st century [1]. Therefore, there is growing interest for interventional and observational studies already in pre-school children (2-6 years) [2]. However, such studies are difficult to conduct since traditional dietary assessment methods have limited accuracy, and involve excessive effort for caretakers and researchers [3]. Mobile phones open new possibilities since they are readily available, enable instant data digitalization, and contain a camera for photographing pre- and post-meal food items. Photos using digital cameras have shown potential for assessing dietary intake in both adults [4-6] and children [7-9]. We have developed a new tool for assessing energy and food intake using mobile phones, the Tool for Energy Balance in Children (TECH). The aims of this pilot study in healthy 3 year olds were (1) to compare energy intake by means of TECH with total energy expenditure (TEE) measured using the doubly labeled water (DLW) method, and (2) to compare intakes of fruits and berries, vegetables, juice, and sweetened beverages assessed by means of TECH with corresponding intakes obtained using the Web-based KidMeal questionnaire (KidMeal-Q), previously validated against a 7-day food record. The selected foods were considered as relevant markers for good (fruits and berries, vegetables, and juice) and bad dietary habits (sweetened beverages).

## Methods

### Recruitment and Protocol

Healthy, 3 year old Swedish children (N=30) were recruited in 2010-2011 [10-12]. Their mean TEE was measured for 14 days using the DLW method [11,12]. During this period, the children’s intakes of foods and drinks were assessed using TECH, and parents completed the KidMeal-Q. A complete data collection was obtained from 30 children. The mean change in body weight between day 1 and 14 was -0.008 kg (SD 0.317). All children originated from a well-educated, middle-income population. The study was approved by the Research Ethics Committee, Linköping, Sweden. Written informed consent was obtained from all parents.

### Tool for Energy Balance in Children (TECH)

**Figure 1:**
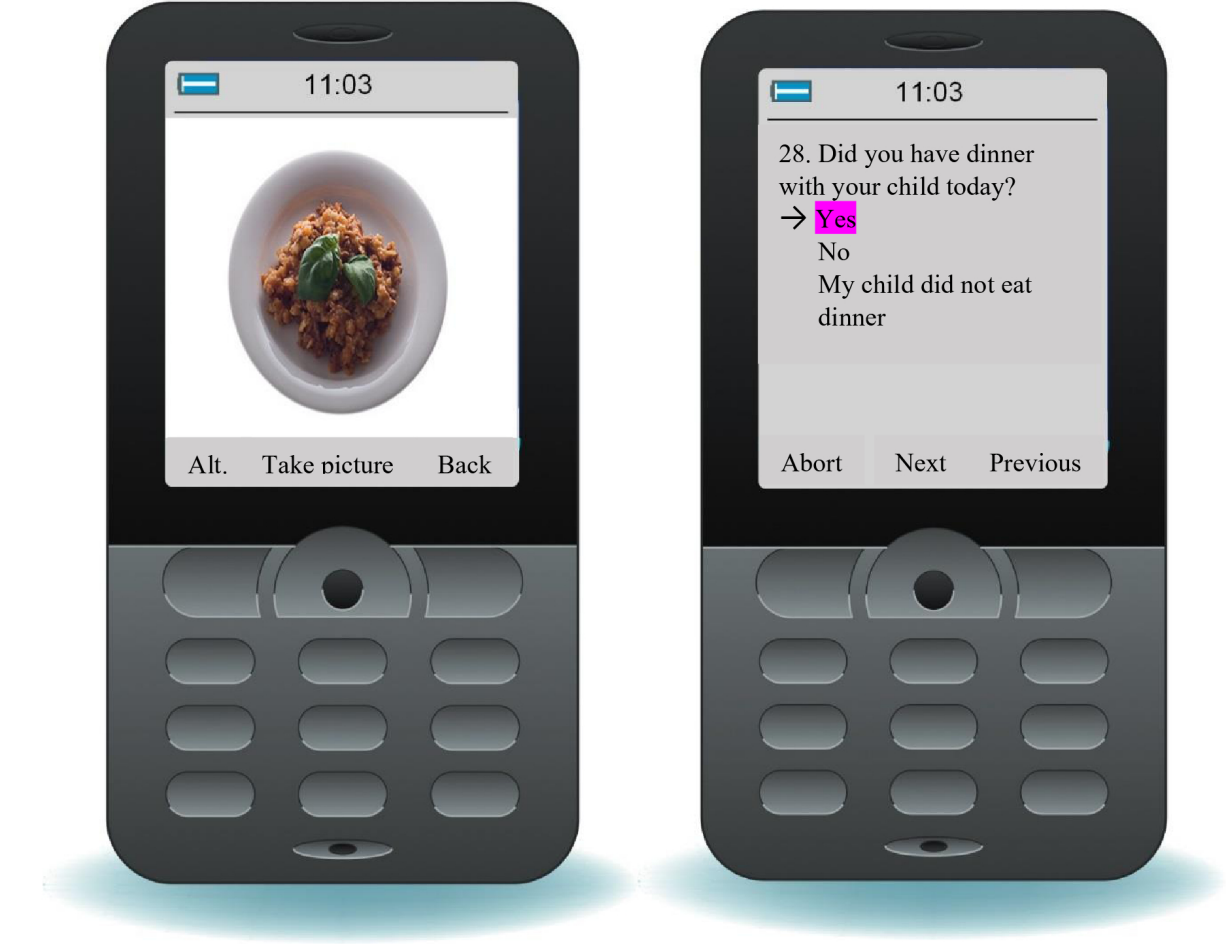
Screenshot of a TECH question and picture.

Parents and other caretakers were instructed to take pre- and post-meal photographs of all the food items and beverages consumed by their child during one 24-hour period using a mobile phone provided for the study. At each meal, they also answered 6-7 questions regarding the type of milk, butter/margarine/oil, meat, bread, cereal, and sauce using a JAVA-based questionnaire installed on the mobile phone (see Figure 1). The parents were instructed to photograph the meals from three angles, to use tableware provided specifically for the study, and to place a matchbox in each image. Volumes of foods were assessed from images using known sizes of the tableware and matchbox identifications by means of the software Paint (Microsoft, version 6.1), and converted into weight by being multiplied by the appropriate weight per volume [13]. The energy intake was calculated from intakes of foods and drinks through linkage to the Swedish Food Database [14].

### KidMeal-Q

**Figure 2:**
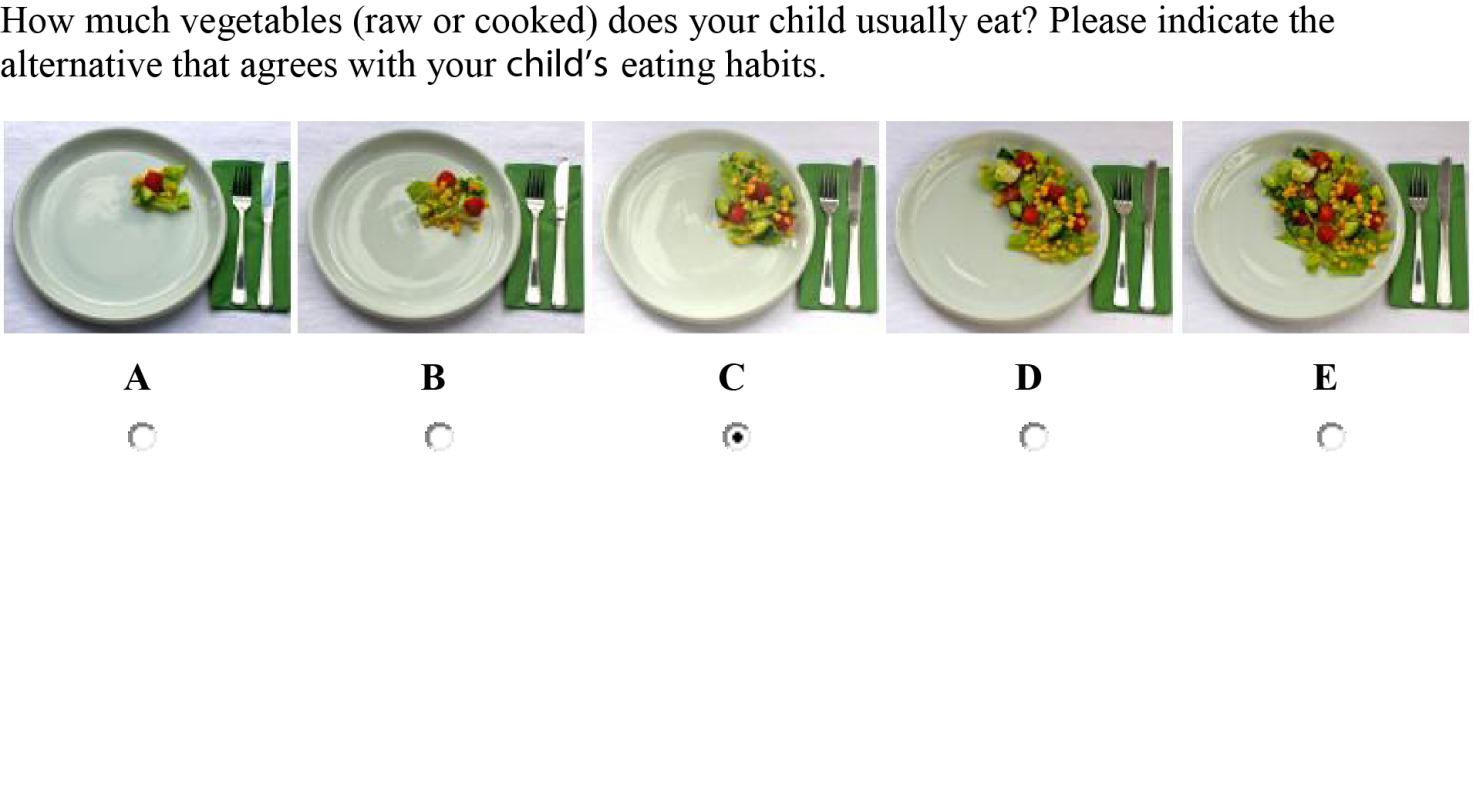
Screenshot of a sample KidMeal-Q question.

KidMeal-Q was developed in 2008 for the LifeGene study [15]. It is an online, meal-based food frequency questionnaire designed for children aged 3-5 years for assessing dietary intake during previous months, and covers 42-86 food items, drinks, and dishes, depending on the number of follow-up questions (see Figure 2). For each child, we converted the reported frequency for vegetables into daily intakes by multiplying them by the reported portion sizes (using six pictures). KidMeal-Q does not provide any portion sizes for juice, fruits, berries, or sweetened beverages, and thus standard portion sizes [14] were used to convert reported frequencies into daily intakes.

KidMeal-Q was validated against a 7-day weighed food record in 23 healthy Swedish children with a mean age of 4.6 years (SD 1.5), weight of 18.4 kg (SD 3.7), and height of 1.09 m (SD 0.11) (data to be published). In that study, correlation coefficients between intakes of vegetables, fruits, and juice to sweetened beverages assessed using KidMeal-Q and food record estimates were .45 (*P*=.03), .59 (*P*=.003), and .53 (*P*<.001), respectively. These correlation coefficients are similar to those reported for adults when comparing food-frequency questionnaires with food records [16,17]. Therefore, although not an established reference method, we consider KidMeal-Q to be an appropriate reference in this first evaluation of TECH.

### Statistical Analyses

Values are given as means (SD). Significant differences between mean values were identified using the Wilcoxon matched pairs test. Correlation analyses were performed using Pearson or Spearman rank order correlations. The Bland-Altman procedure [18] was used to assess the agreement between methods. Analyses were performed using Statistica Software, version 10 (STAT SOFT, Scandinavia AB, Uppsala, Sweden).

## Results

The mean age of the children in the study was 3.00 years (SD 0.04), with a mean weight of 15.4 kg (SD 1.6), height of 0.96 m (SD 0.03), and body mass index (BMI) of 16.6 kg/m^2^(SD 1.2). Five children were classified as overweight, and none as obese [19].

**Figure 3:**
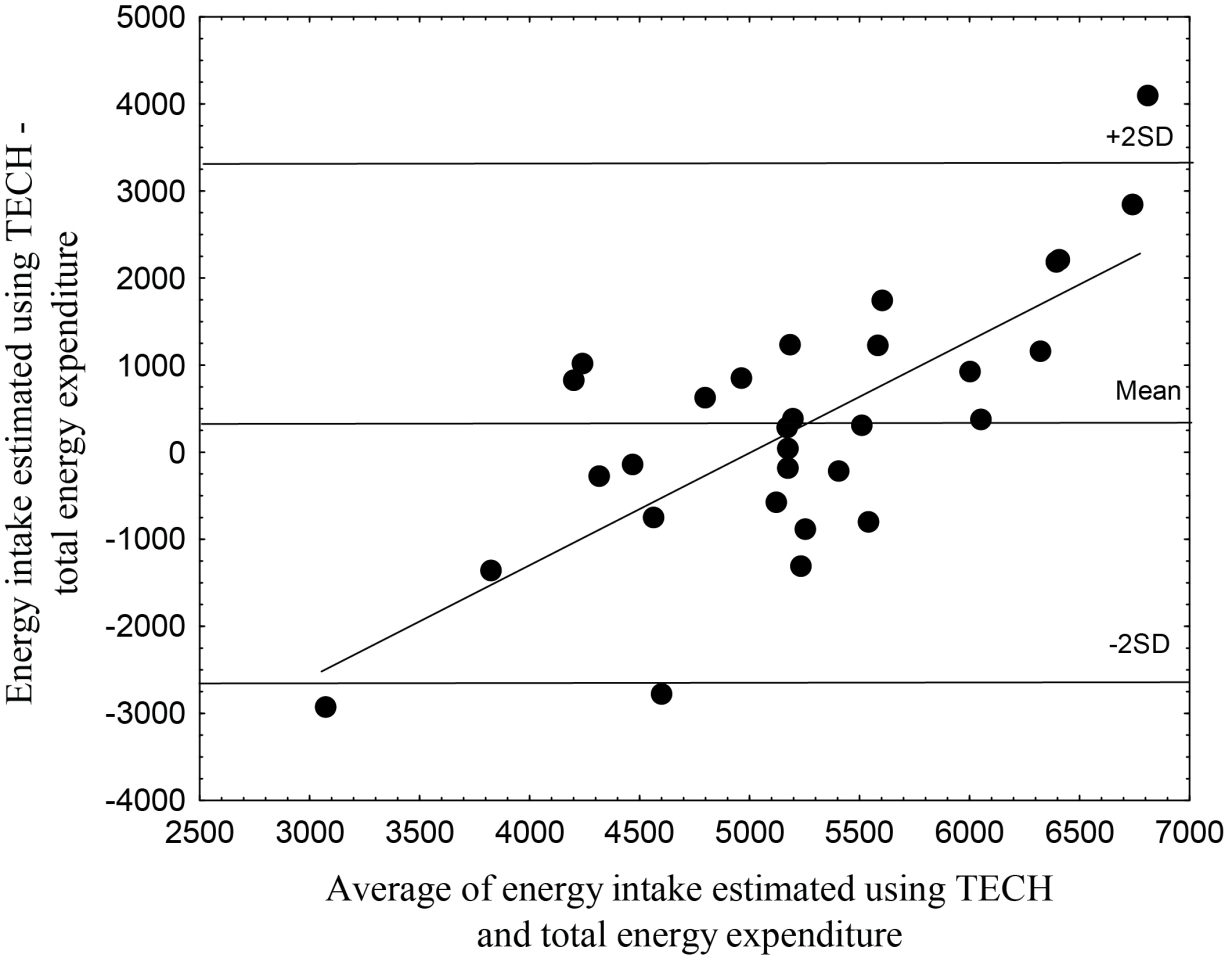
A Bland-Altman plot comparing energy intake estimated using TECH and total energy expenditure measured using the doubly labeled water method in 30 healthy 3 year old children. The mean difference between the methods was 330 kJ/24h with limits of agreement (2SD) of 2990 kJ/24h [16]. The regression equation was y=1.27(x−6312.67) (*r*=.73, *P*<.001).

The mean energy intake, assessed using TECH (5400 kJ/24h, SD 1500) was not significantly different (*P*=.23) from TEE (5070 kJ/24h, SD 600). Figure 3 shows the Bland-Altman plot for energy intake compared to TEE. The limits of agreement were wide, and TECH overestimated high energy intakes and underestimated low energy intakes.

The mean daily intakes of fruits and berries, vegetables, juice, and sweetened beverages using TECH and KidMeal-Q are shown in Table 1. No significant differences between the two methods were observed. When comparing intakes of fruits and berries, vegetables, juice, and sweetened beverages using TECH with the corresponding KidMeal-Q estimates, the Bland-Altman plots were similar to the corresponding plot for energy intake (ie, they showed large limits of agreement and trends toward an overestimation of high intakes and an underestimation of low intakes, figures not shown). Furthermore, significant correlations between the two methods were observed for intakes of fruits and berries, vegetables, and juice (Table 2).

**Table 1:** Mean daily food intake estimated by means of TECH andKidMeal-Q (N=30).

Food group	TECH^a^		KidMeal-Q^b^		*P* ^d^
	Intake (g/day^c^), mean (SD)	Range (g/day), min-max	Intake (g/day), mean (SD)	Range (g/day), min-max	
Fruits and berries	89 (110)	0-309	88 (31)	37-125	.636
Vegetables	40 (52)	0-255	40 (36)	0-120	.568
Juice	50 (79)	0-240	59 (64)	0-300	.243
Sweetened beverages	95 (120)	0-320	36 (30)	0-96	.061

^a^Tool for Energy Balance in Children

^b^KidMeal Questionnaire

^c^grams/day

^d^ *P* value for difference between daily intake estimated by means of TECH and KidMeal-Q using the Wilcoxon matched pairs test

**Table 2:** Correlation coefficients between food intakes (grams/day) estimated using TECH and KidMeal-Q (N=30).

Food group	*ρ* ^a^	*P*
Fruits and berries	.46	.010
Vegetables	.43	.020
Juice	.42	.020
Sweetened beverages	.15	.424

^a^Spearman rank order correlation

## Discussion

### Principal Findings

This is the first study that has evaluated energy intake assessed using mobile phones versus the DLW method in preschoolers. The average energy intake estimated using TECH was not significantly different from the average TEE (mean difference +7%). However, the limits of agreement in the Bland-Altman plot were wide, indicating low accuracy for TECH in estimating energy intake for individuals. Furthermore, we observed a bias where high energy intakes were overestimated while low energy intakes were underestimated. Although we only used TECH for one day, the mean difference and our limits of agreement for energy are comparable to previous validation studies for established dietary methods in children aged 3-6 years [20-25]. In our study, part of the inaccuracy for individuals may be the result of TECH being applied for only one day.

There is no reference method for intakes of foods and drinks. Hence, as commonly applied, we compared TECH with another dietary method to evaluate its relative accuracy for foods. Despite that TECH was only applied for one day, which may not represent habitual intakes, we obtained correlations for fruits and berries, vegetables, and juice with KidMeal-Q (which assesses habitual intakes in past months) that were of similar magnitude as those commonly reported when comparing two dietary methods [16,17]. Although the accuracy in individual children was low, the average intakes of fruits and berries, vegetables, and juice assessed using TECH were comparable to corresponding figures with KidMeal-Q. However, our results for foods need confirmation due to the different assessment periods.

### Study Strengths and Limitations

The major strength of our study is that we compared energy intake to TEE using the DLW method, which is the gold standard when validating reports of energy intake [3,26]. This is superior to using another dietary method as a reference since all such methods are well known to be associated with systematic errors [3,27]. The DLW method can be used as a reference since energy intake and TEE should be equal for subjects in energy balance during the measurement period. This criteria is valid both in normal-weight and overweight subjects, and was fulfilled since our children were weight stable through the 14-day-period, and since the energy content of retained tissue corresponds to only approximately 1% of energy intake at this age [28,29]. A limitation is that we compared one-day TECH data with mean TEE data from 14 days. However, this has unlikely influenced our results since the day-to-day variation in TEE is small [30,31].

The major limitation of this pilot study is that we applied TECH during only one day. The reason for this is that this first evaluation of TECH was conducted within an on-going study and we did not want to affect the parents’ participation in the original study by adding more assessment days. Furthermore, our sample size was small and participating families represented a selected group, which may limit generalizability. However, since the accuracy of using TECH for one day was comparable to established dietary methods, future research should evaluate if the accuracy for TECH can be improved with more days. In addition, identification of the underlying reasons for the observed bias in TECH could be the topic for future studies.

### Conclusion

In conclusion, one day of recordings using TECH is not able to accurately estimate intakes of energy or certain foods in 3 year old children.

## Abbreviations

TECH: Tool for Energy Balance in Children
DLW: Doubly labeled water
TEE: Total energy expenditure
KidMeal-Q: KidMeal questionnaire
